# An Experiential Approach to Training Medical Faculty to Coach: “The Total Experience was Much More Than the Sum of Its Parts”

**DOI:** 10.1007/s10880-024-10038-0

**Published:** 2024-08-23

**Authors:** Binata Mukherjee, Rebecca Smith, Gurupreet Khalsa

**Affiliations:** 1 Faculty and Professional Development & Internal Medicine, Frederick P. Whiddon College of Medicine, University of South Alabama, CSAB 104, 5795 USA Drive North, Mobile, AL 36688-0002 USA; 2 Mitchell College of Business, University of South Alabama, Mobile, AL USA; 3 Frederick P. Whiddon College of Medicine, University of South Alabama, Mobile, AL USA; 4 Department of Counseling and Instructional Sciences, College of Education, Program of Instructional Design and Development, University of South Alabama, Mobile, AL USA

**Keywords:** Coach training, Experiential learning, Self-determination theory, Intentional change theory, Academic medical center

## Abstract

Coaching in academic medicine may be used for performance improvement as well as personal and professional growth and development. Medical faculty used to advising and mentoring learners may find it challenging to transition to coaching. Limited information is available about educating physicians to take on the role of coaching. We investigated a faculty coach training program at an academic medical center, using qualitative methods to explore how participants’ perceptions of the training aligned with the elements of Self-Determination Theory (SDT) and Intentional Change Theory (ICT) that were taught using the principles of Experiential Learning Theory (ELT). Based on findings that illuminated understanding and practice of coaching, it may be summarized that the application of experiential learning may be an effective approach in helping faculty embrace the principles of SDT and ICT and make the shift to transformational coaching.

**Supplementary Information:**

The online version contains supplementary material available at 10.1007/s10880-024-10038-0.

## Introduction

‘Coach,’ meaning carriage, is a metaphor for carrying a coachee from one state to another. International Coach Federation (ICF), the leading global, independent, credentialing entity for practitioners, defines coaching as “partnering with clients in a thought-provoking and creative process that inspires them to maximize their personal and professional potential” (International Coaching Federation, n.d.). Margaret Moore, co-founder of the Institute of Coaching (IOC) which is dedicated to bringing science to coaching, defines coaching as a “growth-promoting relationship that elicits autonomous motivation, increases the capacity to change, and facilitates a change process through visioning, goal-setting, and accountability which at its best leads to sustainable change for the good” (Moore et al., 2016, p. 1). Coaching adapted for medicine is “the art and science of facilitating sustainable change and growth of medical students and residents to realize their full potential as master adaptive learner. Key areas…include optimal learning, well-being, professional development and performance, and leadership” (Hammoud et al., 2023, p. 20).

Two fundamental uses of coaching have emerged across sports, music, business, and medicine: performance, or skill-based, career coaching (Spatz et al., 2021; Stephany et al., 2023), particularly during role transitions, and developmental or transformational (Marcdante & Simpson, 2018; Schulte et al., 2019). The essence of coaching, individual or group, hinges on change centered in relationship or partnership, goal-setting, and accountability through reflection by the coachee (Hammoud et al., 2023; Passmore & Lai, 2019; Radha Krishna et al., 2019). The relationship in the coach–coachee dyad stimulates internal motivation facilitating change through visioning, self-discovery, and self-knowledge. Key elements of the process include (a) learner focus, (b) trusting relationship, (c) introspection, and (d) mirroring back to the coachee (Hauer et al., 2022; Orr & Sonnadara, 2019; Vandaveer et al., 2016). Unconditional positive regard is foundational for a strong coaching alliance (Athanasopoulou & Dopson, 2018). Coaching is a social process whereby the coach and coachee influence each other and are influenced by their mutual multidimensional contexts; building trust in the partnership becomes critical, especially in a medical learning environment where hierarchies have dominated (Hauer et al., 2022; Watling & Ladonna, 2019).

The need to acquire tremendous amounts of factual knowledge, compounded by legal and governance issues, has led to an emphasis on unidirectional didactic physician training (Launer, 2018). Thus, physicians typically advise and mentor (Santiesteban et al., 2022), instead of encouraging interactional self-discovery integral to coaching (Tippit et al., 2024). Advising is providing answers to questions brought in the conversation, while mentoring entails a relationship between an experienced individual and a young professional or learner embarking on their career (Secchin et al., 2020). Advisors are expected to be experts, while mentors not only provide expertise but also share their experience in navigating a junior’s envisioned path (Hammoud et al., 2023). A skilled coach, especially a developmental coach, need not be an expert in the topics the coachee wants to explore. All three methods are meant for improvement (Louridas et al., 2022), employ common skills (relationship, trust, listening, reflection), and often the same faculty participate in all these roles. The terms are often used interchangeably in academic medicine leading to confusion instead of distinctions among them (Marcdante & Simpson, 2018; Orr & Sonnadara, 2019; Radha Krishna et al., 2019). The roles can be mapped on a continuum of improvement and not pitched against each other (Hammoud et al., 2023; Louridas et al., 2022). Novice learners requiring skills acquisition may benefit from advising, while becoming an expert may call for non-directive self-reflection with a coach. Both mentoring and coaching may be used for professional or personal growth and development.

Traditionally, coaching is used in academic medicine for learning performance-related skills, including surgical skills (Gawande, 2011), competency-based medical education (Lovell, 2018; Orr & Sonnadara, 2019), improved communication (Frates et al., 2011; Stephany et al., 2023), and professional skills. Transactional skills acquisition generally involves direct observation, self-assessment and reflection, feedback, and goal-setting (Chen et al., 2021; Graddy et al., 2018; Orr & Sonnadara, 2019). Due to rapid changes in medicine, learning extends beyond school and is a continuous professional development process. Hence, transformational coaching focusing on intrinsic needs (Orr & Sonnadara, 2019) might be an appropriate application of coaching in medical education (Deiorio et al., 2016). Emerging transformational coaching in academic medicine promotes professional identity formation for learners (Parsons et al., 2021; Wolff et al., 2019), deepening of physicians’ own identity as a result of coaching (Branzetti et al., 2023; de Lasson et al., 2016), and development of self-awareness, self-determination, and self-monitoring skills critical to lifelong learning in actualizing their highest selves (Dyrbye et al., 2019; LCME, 2023; McGonagle et al., 2020). Considering that coaching helps in resilience and well-being (Brooks et al., 2020; Gazelle et al., 2015; Stephany et al., 2023), it may be confused with psychotherapy; while coaching is present and future oriented, psychotherapy delves into the past to resolve current challenges (Passarelli et al., 2024). Coaching also helps in addressing disruptive behavior (Hammoud et al., 2023; Love et al., 2021) and remediation (Lovasik et al., 2022; Stephany et al., 2023). Coaching may address both performance and developmental needs simultaneously (Parsons et al., 2021). Coaching methods are context- and need-dependent, from directive, autocratic, dictating behavior to democratic, shared decision-making (Orr & Sonnadara, 2019), with the latter generating more insights (Moore, 2023b); thus no “true” coaching methodology exists. With the increasing use of coaching in medicine, institutions are creating programs to achieve goal-appropriate outcomes to benefit all stakeholders. We focused on transformational coaching rather than skills acquisition.

While coaching might elicit self-actualization, not everyone is ready for transformation requiring internal motivation and ownership of the developmental process (Raihan & Cogburn, 2023). Implementation of coaching also necessitates access to qualified coaches. Coaches can be external and cost-intensive or internal, requiring training faculty to coach (Passarelli et al., 2024), which may not come naturally for physicians (Orr & Sonnadara, 2019). Watling and LaDonna (2019) found that medical faculty struggled with developing as coaches and experiencing vulnerability. Faculty development addressing the underlying tension between facilitative and teaching roles becomes critical (Launer, 2018).

Passarelli et al. (2024) highlighted that “research on coaching for physicians is in its infancy” (p. 783). Few studies have focused on the process of training faculty to coach (Kopechek et al., 2017; Thorn & Raj, 2012; Watling & LaDonna, 2019). Most coach training programs are short, didactic (Palamara et al., 2015; Price et al., 2021), and often part of general faculty development (Bakke et al., 2020; Elster et al., 2021; Hall et al., 2012) rather than coaching specific (Tippit et al., 2024). While existing resources including online content are beneficial and accessible, they do not address holistic learning encompassing essential deliberate coaching practice (Orr & Sonnadara, 2019; Tippit et al., 2024). A significant gap exists in the literature regarding training of faculty to transition from advising and mentoring (Orr & Sonnadara, 2019) to coaching (Gonzalo et al., 2019), a potentially arduous and uphill task for physicians used to advising learners and patients. Studies have emphasized the importance of training (Armson et al., 2019; Gonzalo et al., 2019; Hall et al., 2012) for faculty coaches and evaluating the quality of such training to ensure that the coaching provided by faculty meets the defined program goals.

We believed that transformational coaching may be best understood by both teaching and faculty experiencing Deci and Ryan’s (1985) self-determination theory (SDT) and Boyatzis et al.’s (2013) intentional change theory (ICT) during the training. Self-determination is a person’s ability to reach their highest level of motivation, engagement, performance, persistence, and creativity, provided a nurturing environment supports three psychological needs: autonomy, competence, and relatedness. Helping physicians shift to of self-determination for the learner during coaching would afford an opportunity to foster rapport and to fully engage in coachees’ motivations and strengths. The leadership coaching model ICT entails self-discovery of one’s ideal and real self, creating a learning path, and experimenting with new practices leveraging the coaching relationship. Self-development rests on internal motivation, and use of metacognition, awareness about one’s thinking, to plan learning following recognition of gaps; thus, SDT and ICT were considered appropriate theories to help faculty understand the coaching process.

According to Kolb’s Experiential Learning Theory (ELT) (Kolb, 2014), learning is “the process whereby knowledge is created through the transformation of experience” (p. 93). The combination of grasping and transforming experiences results in knowledge. Grasping, the process of taking in information, arises from concrete experience and abstract conceptualization, while transforming, how one interprets and acts on information, includes reflective observation and active experimentation. Reflection allows one to exercise authority over thoughts and beliefs (Gazelle et al., 2015) in an intelligent self-dialogue (Launer, 2018); therefore, reflecting on coaching practice during training would increase awareness of skills. Irrespective of the type of coaching, reflection is foundational (Orr & Sonnadara, 2019). Deliberate practice in a safe environment facilitates acquiring new habits before implementing in the real world. Learning is continuously created in a recursive manner by variably using the four elements experiencing, reflecting, thinking, and acting of the learning cycle. Bringing awareness to the process enhances learning. The emphasis of the training was therefore on using these phases as coaches and coachees. The purpose of the study was to examine participants’ descriptions of coach training that highlighted their understanding of transformational coaching, coaching principles of SDT and ICT, and the application of ELT concepts.

## Method

### Study Design

The college of medicine (COM) where this study took place had 290 medical students, 263 residents, 33 fellows, and approximately 200 faculty in 2021. All faculty were invited through a Qualtrics survey and direct email for voluntary participation in a coaching training program to become coaches.

Our approach was a social constructivist stance respecting participants’ perspectives and acknowledging that reality is subjective for every individual (Creswell & Poth, 2018). We used a deductive consensual thematic analysis to understand perceptions of faculty learning to coach in accordance with SDT and ICT and how their learning experiences aligned with components of ELT. Participants were not asked about the coaching theories or the principles of ELT. Instead, we triangulated data from three sources for discovery of the understanding of the elements of SDT and ICT and application of ELT. The University’s Institutional Review Board approved the study as exempt. All participants consented to their views being included in the study.

### Coach Training Program

In the absence of established core competency requirements for physician coaches or available physician coach training curriculum, this program was designed by BM based on her coach and mentor training, and coaching practice (Mukherjee et al., in press). As an experienced educator, she incorporated pedagogical best practices including elements of ELT (Kolb, 2014) for engaging participants. ICF’s core competencies were the coaching standards because it is the most widely used, the trainer BM is ICF trained, and academic medicine coaching competencies had not been published (Passarelli et al., 2024; Wolff et al., 2021).

Faculty motivated to devote the time necessary to complete the training attended. White women comprised the majority of the 20 participants reflecting the racial diversity of the COM. The 34 h program (Table 1) implemented from May to November was designed to achieve several objectives. During 26 h of training sessions, faculty identified and practiced coaching skills and ICF coaching competencies and were introduced to the Master Adaptive Learner (MAL) concepts (Cutrer et al., 2017). A further 8 h were devoted to discussions about a future academic medicine coaching program.

**Table 1:** Session details of the coach training program at an academic medical center

No	Topic	Readings	Visuals	Assessments
*Core principles of academic coaching*				
1	The master adaptive learner	Cutrer et al. (2017) Christensen et al. (2020)	Zander (2012)	Five-dimensional curiosity scale (Kashdan et al., 2018) Mindset (Dweck, 2016) Groningen Reflection Ability Scale (Andersen et al., 2014) Connor–Davidson Resilience Scale (n.d.)
2	Foundation of coaching^a^	Lovell (2018) Boyatzis et al. (2013) Gawande (2011)	Trainer being coached^b^	Rational-Experiential Inventory (Pacini & Epstein, 1999)
3	Coaching to prepare the master adaptive learner^a^	None	Film (Redford, 2000)	None
4	Self as a coach	Neenan (2009)	None	None
5	Self-awareness	Davis et al. (2006)	None	EQi 2.0 (n.d.)
6	Growth and development through strength	None	None	VIA Survey of Character Strengths (https://www.viacharacter.org/)
7	Building trust	Najibi et al. (2019)	None	None
8	Coaching feedback^c^	None	None	None
9	Coaching program example	Wolff et al. (2019)	Speaker^d^	None
*Designing, implementing, and evaluating an academic coaching program*				
10	Vision and desired goals for UME Coaching Program	Parsons et al. (2021)	None	None
11	Vision and desired goals for GME Coaching Program Faculty development and scholarship on coaching	None	AMA speaker	None
12	Structure and process	Palamara et al. (2018)	None	RACI matrix (Jacka & Keller, 2009)
13	Resources and evaluation	None	None	Logic model [Discussion]
14	Conclusions Graduation ceremony	None	None	End-of-program survey [Program evaluation]

^a^Coaching models: (1) GROW (goal (G), reality (R) of the current state, options (O) to reach the destination, and way (W) forward), (2) WOOP (wish (W), desired outcomes (O), obstacles (O), and a plan (P) to overcome obstacles), and (3) SMART (specific, measurable, achievable, relevant, and timely) goals (Moore, 2023a)

^b^Live demonstration of coaching performed by ICF-certified master-level physician coach

^c^Feedback from 1 external observer for each 4 participants

^d^Speaker from an academic medical center that implemented a coaching program

*AMA* American Medical Association, *ICF* International Coaching Federation, *UME* undergraduate medical education (students in the MD program), *GME* graduate medical education (residents and fellows), *RACI* responsible, accountable, consulted, and informed, *VIA* values in action

Lectures were intentionally less than a third of session time to allow reflection, discussion, and coaching practice. Faculty were organized in groups of four to practice coaching each other rotating among coach, coachee, and observer during every session, excluding the first. The trainer moved among groups to provide feedback on performance against ICF core competencies (Appendix A). Practice coaching also occurred in the large group for peers and the trainer to provide feedback. At the end of the training component, ICF-certified coaches provided individual written feedback after observing participants coach. Enabling faculty to be vulnerable to experience coaching and be able to build a working alliance during the training entailed creating a safe and trusting environment, labeled relatedness in SDT (Ryan & Deci, 2019). In accordance with ICT, evoking self-knowledge is inherently difficult unless sequenced on visioning the ideal self, followed by the real self (Jack et al., 2023), which was integral to this training. During practice, faculty coachees were brought to a positive state of mind for feeling safe and building trust, followed by visioning, evoking awareness, and designing action plans toward vision realization. Goal-setting models considered helpful in fostering self-directed learning and intrinsic motivation were discussed. Attendees reflected and discussed using Pear Deck (https://www.peardeck.com/) during, and Ment.io (not in business) between, sessions.

Considering the relevance of curiosity, reflection, growth mindset, resilience, and emotional intelligence for coaching and to facilitate experiencing expectations of learner coachees, faculty completed validated assessments of curiosity (Five-dimensional Curiosity Scale) (Kashdan et al., 2018), resilience (Connor–Davidson Resilience Scale) (Connor & Davidson, n.d.), emotional intelligence (EQi 2.0: https://www.multi-health-systems-usd (mhs.com)), innate preference of processing information (Rational-Experiential Inventory 40) (Pacini & Epstein, 1999), character strength (Values in Action: https://www.viacharacter.org/character-strengths), and reflection ability (Groningen Reflection Ability Scale) (Andersen et al., 2014). An instrument assessing mindset (Dweck, 2016) was used. They interpreted and used the results to coach each other. Participants received continuing medical education credits and a certificate of completion.

### Data Collection

We collected data from three sources (Appendix B): (a) reflections between sessions, (b) anonymous post-training survey using scaled and open-ended questions through Qualtrics, and (c) feedback during three focus groups, approximately 90 min long, attended by 11 participants. Focus groups were facilitated by two individuals uninvolved with the training to minimize potential bias, using a semi-structured interview protocol. The videoconference software Zoom provided a verbatim transcript of the conversations. A graduate assistant who was neither a trainer nor a facilitator edited and compiled the transcripts. Transcriptions were confirmed with participants to ensure accurate representation of their viewpoints before deidentification. The scaled questions explored change in understanding of coaching, differences among advising, mentoring and coaching, and the perceived value of the training.

### Data Analysis

We employed an iterative deductive consensual thematic approach to coalesce disparate elements of the data through inquiry into participants’ experiences of the training and attitudes about being a coach. The authors reflected and acknowledged how their background, experience with coaching and faculty development, and stance may color analysis and interpretations. BM, the primary researcher, is a physician administrator and a coach involved with faculty and professional development in the COM. RS is a mental health therapist serving students and faculty in the COM. GK is a qualitative researcher and educator.

BM and GK independently and recursively extracted important statements and key phrases from all three data sources. BM tabulated the data using Microsoft Excel and categorized according to a priori codes based on the elements of the coaching theories, namely autonomy, competence, relatedness/relationship, ideal self, real self, learning path, and practice. These codes were then cross-tabulated with the experiencing, reflecting, thinking, and acting phases of ELT. We discussed and examined how the statements aligned within and across categories to ensure that the elements of all three theories represented the entire dataset. Results from the scaled questions were tabulated as counts.

## Results

Analysis of qualitative data from all three sources using a priori codes derived from the theories revealed strong alignment of participants’ experiences with all the seven elements of SDT and ICT, as well as the four phases of ELT used as instructional method (Table 2). Further, the post-training survey results (Table 3) revealed that the understanding of coaching had increased for all respondents (N = 18) even though initially faculty stated they “didn’t understand what coaching was.” Seventeen (94%) indicated improved attitude about coaching. Awareness of the distinctions among advising, mentoring, and coaching, as well as the benefits of coaching were reported: “the total experience was much greater than the sum of its parts.”

**Table 2:** Total instances of elements of SDT, ICT, and ELT from all data sources

Source	Components of experiential learning theory (ELT)				Components of self-determination theory (SDT)/intentional change theory						
	Experiencing	Reflecting	Thinking	Acting	Autonomy	Competence	Relatedness/relationship	Ideal self	Real self	Learning path	Practice
Focus groups	51	43	35	19	19	40	21	7	12	24	14
Post-training survey	16	8	7	7	2	9	0	1	3	17	6
Reflection journal	0	99	42	0	45	45	14	26	3	17	6
TOTAL	67	150	84	26	66	94	35	34	18	58	26

**Table 3:** Participants’ responses to select post-training survey questions highlighting understanding of coaching

Thinking about before and after the program, please select the response that is most appropriate for you					
	Significantly decreased	Somewhat decreased	Neither decreased nor increased	Somewhat increased	Significantly increased
My understanding of coaching	0	0	0	0	18
My understanding of the difference between coaching, mentoring and advising	0	0	0	1	17
My attitude toward coaching	0	0	0	1	17
My belief in benefits of coaching	0	0	0	1	17
On a scale of 1 to 10, 1 being not at all and 10 being extremely likely:	1 or 2	3 or 4	5 or 6	7 or 8	9 or 10
How likely are you to recommend the Academic Coaching Program to a colleague?	0	0	0	1	17
	Strongly disagree	Disagree	Neither disagree nor agree	Agree	Strongly agree
Overall, I found the program valuable	0	0	0	1	17

### Autonomy

Faculty underscored the powerful effects coaching might have on learners in assisting them to achieve personal and professional growth, provided they relinquish control of the coachees’ developmental needs and give them the autonomy of determining what those needs were and how they would be met. The realization came through reflecting, thinking, and acting principles of ELT, as expressed in statements like “it meant shut up” and “sometimes we are patriarchal but it doesn’t have to be that way.” Faculty expressed recognition that coaches were expected “to be able to actually facilitate people sorting things out on their own.” One participant summarized their understanding of coaching as “more kind of metaphysical but really, it’s to give people, the coach, the tools to help the coached discover things on their own.”

### Competence

Competence of SDT was contextualized as coaching skills for faculty to uncover learners’ needs; “one thing I learned about coaching is that…it opens up your eyes to a lot of things not just about yourself but also how you can help other people.” Multiple participants commented that the training helped them feel competent to coach: “we all grew in our knowledge from maybe a basic definition to really understanding the process.” Participants discovered the inadequacy of “problem-solving,” noting the fluidity in the coaching process in order “to let there be some silence…that was the hardest part.” Becoming competent at coaching was facilitated by becoming reflective and self-aware rather than focusing solely on outcomes.

### Relatedness/Relationship

In SDT, the third psychological need is relatedness, labeled relationship in ICT. During the training, where it felt safe to be vulnerable, participants experienced social cohesion, a trusting “sense of community,” that was deemed invaluable. Faculty appreciated the support they received: “people interested in my well-being and the well-being of the peers was nice because it was rough during the COVID delta surge.” Even colleagues who had known each other for years deepened relationships, “having established that it was safe amongst ourselves was to me pretty priceless.” An empathetic relationship contributes to wellness by allowing the coachee to feel seen and understood. A participant observed, “Compassionate coaching…makes me reflect on the way I approach challenging situations in resident education. Showing care and concern for the other person [shows] a relationship based on trust can be built, helping the resident achieve his/her full potential.” Being non-judgmental, asking questions, and active listening created a “safe space” which felt intimate. Participants noted that the medical hierarchy was “destroyed” by learning together and switching roles in the training environment. One participant found the experience “transformative.”

### Ideal Self

ICT starts with visioning of the ideal self. Participants experienced, reflected, thought, and started acting on their own ideal self to understand the importance of leading coachees to a vision of their ideal self. Understanding this concept was reflected in,

I want someone to come back and say to us, years from now, that, you know, ‘I became more self-aware. I became more understanding where my strengths and weaknesses were. It allowed me to choose my career path in a much more informed way.’

Some participants identified that a new path as a coach was an actualization of an ideal self: “The piece that fell together for me was to understand my suitability toward being a coach. It answers a calling.” Others noted, “It’s a professional development that actually is a personal development program,” and “It added a lot to me as a person.”

### Real Self

The real self means an awareness about the present state one is in and includes the strengths, weaknesses, the good, and not so good aspects. Participants confessed that reflection was not practiced adequately by physicians: “I claim that I have very little time [for reflection]. We are task masters checking off things to do but we never catch up. A lot of us aren’t well trained at true reflection.” Each session started with a welcomed mindfulness activity that provided an opportunity to disconnect from busy schedules. Faculty appreciated the time to “settle down and meditate and bring our focus back to where we were. And take three deep breaths…I envisioned myself practicing similarly.” The experience of undergoing the training opened participants to new knowledge about themselves that “realization and reflection and knowing yourself better in a certain aspect humbles you.” Opportunities for self-assessments during the training led to an awareness of emotional triggers, “to know that not everybody fits into the same ‘round peg in a square hole’ but they’re going to be different people [with] different attributes.” Many physicians including those in leadership roles were able to translate self-awareness into coaching: “If you can develop a clear vision of who you are and what YOU bring, it will only make you better at doing the same for others.”

### Learning Path

In ICT, the ideal self is actualized through creation of the learning path which alludes to the paradigm shift from advising to coaching and the importance of helping coachees craft their specific learning path. This aspect was realized through experiencing coaching and reflecting. It was noted that “the program showed us what coaching can bring out from within ourselves. Growth was the way to go.” Participants created their own learning path based on their ideal self: “it might be good to spread your wings and get out of your comfort zone.”

### Practice

Practice of ICT was interpreted as faculty’s own practice in learning to coach. Participants’ statements showed how physicians were acting out the skills they were learning in the training: “I actually signed up for this for myself because I wanted better ways to help young faculty…I learned so many things that I can also translate into my everyday work.” One summed up the idea of using coaching in life as “I guess this is just the beginning of my journey.” Participants made a connection between coaching and leadership: “As a leader of a department, I recognize the need to identify opportunities to coach multiple types of people with different career pathways ranging from office personnel, learners to [physicians].”

### Experiential Learning Cycle

Direct employment of the elements of experiential learning, experiencing, reflecting, thinking, and acting (Kolb, 2014) during the training, including guided meditation focused on evoking their vision, facilitated participants’ journeys both as future coaches and for their own personal and professional growth. One participant mentioned that they “greatly admired [the instructor’s] sense of how much was enough didactic versus ‘it is time to stop and talk or act or do.’” The entire experience of the training, including being observed by professional coaches was positive: “[it] wasn’t a lecture on coaching but it was very interactive” and “definitely her teaching tools were terrific. She had so many ways to keep us engaged and interacting.”

## Discussion

Overwhelming interest in coaching learners is a recent phenomenon due to the shift in medical education from informational input to facilitated discursive individual learning (Launer, 2018). Coaching’s “bottom-up” approach (Tippit et al., 2024) entails iterative self-awareness, self-determination, and self-monitoring by the coachee through a process of reflection enabled by a coach (Schulte, 2023; Vandaveer et al., 2016). Our findings align with Passarelli et al.’s (2024) emphasis on coaching theory and science for development of coaches. In institutions implementing or considering coaching for learners, the responsibility of encouraging reflection falls on the shoulders of faculty coaches who are used to advising and mentoring. Despite the need for faculty education addressing the paradigm shift, we could find no faculty coach training program bridging this gap. Our endeavor was to address this need through the coach training program. Based on the results of this study, it may be surmised that experiential learning may facilitate the paradigm shift to coaching. Participating faculty expressed improved understanding of coaching and competence to coach. They underscored that being a successful coach encompasses building a working alliance through a strong relationship and relatedness with the coachee, facilitating the coachee to envision their ideal self as a successful professional and its meaning for each individual. Faculty coaches learned how to question with curiosity and evoke reflective self-awareness in the coachee to enable the recognition of their real self (Bachkirova, 2016; Schulte, 2023; Vandaveer et al., 2016). They also realized the importance of providing autonomy to the coachee in selecting the appropriate learning path among various options for actualizing their physician identity (Schulte, 2023).

With regard to the SDT and ICT elements of relatedness and relationship, respectively, our findings aligned with literature emphasizing the necessity of a trusting environment in which individuals feel safe to show vulnerability (Hauer et al., 2022; Schulte, 2023; Thorn & Raj, 2012). People sharing experiences, an element of ELT, during the training developed bonds that transcended hierarchies, traditions, roles, or environments, which was brought to light by participants in our study. Participants recognized how vulnerability which physicians may not easily embrace (Watling & LaDonna, 2019) helped mitigate existing hierarchical structures (Hauer et al., 2022; Stephany et al., 2023), thereby fostering a sense of community in the training environment.

Although acquiring coaching knowledge is important, the development of the “self as an instrument” of coaching (Bachkirova, 2016, p. 146) may be best achieved through use of the four phases of experiential learning. Using real-life situations alternating as coach and coachee, participants learned to ask non-judgmental questions using a lens of compassionate listening to guide faculty coachees in visualizing goals and in exploring strengths to craft a learning path according to ICT (Boyatzis et al., 2013). Reflecting on the experience of understanding the coaching theories during training provided opportunities for faculty coaches to find their individual learning path for an anticipated shift away from advising (Moore, 2023a). Reflection during coaching practice also provided a better understanding of themselves as evident in our findings. The faculty coaches were able to use the thinking element of ELT to crystallize their thoughts with regard to how coaching may help them in their present work and how a coaching program may be implemented for our learners. Aligned with the acting element of ELT, many faculty coaches immediately started using coaching conversations with students and during clinical rotations, thus putting what they learned into practice.

Analysis of the data highlighted how SDT/ICT helped participants on their path to become transformational/developmental coaches and aligned with Orr and Sonnadara’s (2019) recommendations. Tippit et al. (2024) used experiential learning as an instructional method for performance coach training, and Orr and Sonnadara (2019) emphasized the need for deliberate practice by faculty endeavoring to become coaches. The goal of our program was not to make faculty experts in the knowledge of the coaching theories but to embrace coaching competencies in an experiential way.

Our study was limited by the small number of participants and a mostly homogeneous sample that precluded investigating cultural or demographic differences. In implementing a similar program at other medical colleges, factors unique to each institution must be considered, such as dedicated time for faculty to experience the training, given scheduling conflicts, and the inclination of faculty to embrace coaching. The training environment requires the trainer to guide faculty through being coachees as well as learning to coach. Our study was about the experience of the training program, and we did not evaluate outcomes of coaching with students. Research on developing a toolkit for experiential training of faculty coaches, in addition to documenting the transfer of learned skills to coaching learners, would benefit the academic medicine coaching field.

Unless coaching is supported by institutional culture (Watling & LaDonna, 2019), any coach training may not succeed in overcoming traditional practices of advising and mentoring. Seeking protected time for faculty to coach learners is under discussion in the context of a pilot coaching program for medical students in our institution. Participants’ feedback suggested minor training program changes that included offering the ‘trust’ session earlier and a more structured reflection, which have been incorporated. Through the faculty development office, we intend to recruit more faculty for training and creating a coaching community that will offer opportunities to practice as well as share obstacles and resources.

## Conclusion

This study showed how participants in our coach training program experienced key components of coaching theories using an experiential learning approach. Coaching is known to facilitate growth and development of students and residents in becoming autonomous practitioners. Importantly, transformational coaching transcends traditional advising and mentoring. Participants highlighted the value of learning through experiencing, along with the impact of a reflective journey into self-awareness that improved confidence to embody being transformational coaches.

## Supplementary Information

Below is the link to the electronic supplementary material.

Supplementary file1 (DOCX 40 KB)

## Data Availability

The qualitative data from the audio recordings and transcripts from the focus groups, and discussions and postings during the training cannot be shared due to respondent confidentiality concerns. If there are specific questions about the data, the corresponding author may be contacted to potentially provide additional de-identified responses.
