# Supplementary material for: An Experiential Approach to Training Medical Faculty to Coach: “The Total Experience was Much More Than the Sum of Its Parts”
Source: J Clin Psychol Med Settings. 2024 Aug 23;31(4):769–80. doi: 10.1007/s10880-024-10038-0 (PMC11576883; doi:10.1007/s10880-024-10038-0)
Supplement: Supplementary file 1 — Supplementary file1 (DOCX 40 KB) [file 10880_2024_10038_MOESM1_ESM.docx]

Appendix

1. Coaching Observation Worksheet: ICF Competencies

Coach:_______________________________________________

**Brings coachee into positive state of mind:** Yes No

**Competency 3. Establishes Agreement** Yes No

**Competency 2. Coaching Mindset (open, curious, coachee-focused)** Yes No

**Competency 4. Cultivates trust and safety**

*Did you observe the coach demonstrate any of the following behaviors? If so, how?*

1. Demonstrates respect for coachee’s identity, perceptions ________________________________________________________________________________________________________________________________________________________________________________________________________________________________________________________________________________________________________________________
2. Shows support, empathy, compassion and concern. Supports client’s expression of feelings, beliefs, and suggestions. ________________________________________________________________________________________________________________________________________________________________________________________________________________________________________________________________________________________________________________________
3. Demonstrates transparency ________________________________________________________________________________________________________________________________________________________________________________________________________________________________________________________________________________________________________________________

**Competency 5. Maintains presence**

*Did you observe the coach demonstrate any of the following behaviors? If so, how?*

1. Remains focused, observant, empathetic and responsive to coachee ________________________________________________________________________________________________________________________________________________________________________________________________________________________________________________________________________________________________________________________
2. Demonstrates curiosity ________________________________________________________________________________________________________________________________________________________________________________________________________________________________________________________________________________________________________________________
3. Stays present with coachee’s emotions ________________________________________________________________________________________________________________________________________________________________________________________________________________________________________________________________________________________________________________________
4. Comfortable working in a space of not knowing _________________________________________________________________________________________________________________________________________________________________________________________________________________________________________________________________________________________________________________
5. Creates or allows space for silence or reflection ________________________________________________________________________________________________________________________________________________________________________________________________________________________________________________________________________________________________________________________

**Competency 6. Listens to understand**

*Did you observe the coach demonstrate any of the following behaviors? If so, how?*

1. Reflects what the coachee communicated to ensure clarity and understanding ________________________________________________________________________________________________________________________________________________________________________________________________________________________________________________________________________________________________________________________
2. Inquires when there is more to what the coachee is communicating ________________________________________________________________________________________________________________________________________________________________________________________________________________________________________________________________________________________________________________________
3. Notices and explores coachee’s emotions, energy shifts, non-verbal cues or other behaviors ________________________________________________________________________________________________________________________________________________________________________________________________________________________________________________________________________________________________________________________
4. Integrates coachee’s words, tone of voice and body language to determine the full meaning of what is being communicated ________________________________________________________________________________________________________________________________________________________________________________________________________________________________________________________________________________________________________________________

**Competency 7. Evokes Awareness**

*Did you observe the coach demonstrate* ***any*** *of the following behaviors? If so, how?*

1. Challenges the coachee as a way to evoke awareness and insight ________________________________________________________________________________________________________________________________________________________________________________________________________________________________________________________________________________________________________________________
2. Asks questions that help the coachee explore beyond current thinking ________________________________________________________________________________________________________________________________________________________________________________________________________________________________________________________________________________________________________________________
3. Invites coachee to share more about experiences in the moment ________________________________________________________________________________________________________________________________________________________________________________________________________________________________________________________________________________________________________________________
4. Notices what is working to enhance coachee’s progress ________________________________________________________________________________________________________________________________________________________________________________________________________________________________________________________________________________________________________________________
5. Adjusts coaching approach in response to the coachee’s needs ________________________________________________________________________________________________________________________________________________________________________________________________________________________________________________________________________________________________________________________
6. Invites the coachee to generate ideas about how (s)he can move forward and what (s)he is willing and able to do ________________________________________________________________________________________________________________________________________________________________________________________________________________________________________________________________________________________________________________________
7. Supports the coachee in reframing perspectives _________________________________________________________________________________________________________________________________________________________________________________________________________________________________________________________________________________________________________________
8. Shares observations, insights, feelings, without attachment, that have the potential to create new learning for the coachee. ________________________________________________________________________________________________________________________________________________________________________________________________________________________________________________________________________________________________________________________

**Competency 8. Facilitates Growth**

*Did you observe the coach demonstrate any of the following behaviors? If so, how?*

1. Works with the coachee to integrate new awareness, insight or learning into behavior ________________________________________________________________________________________________________________________________________________________________________________________________________________________________________________________________________________________________________________________
2. Partners with coachee while supporting autonomy to design goals, actions accountability measure that integrate and expand new learning ________________________________________________________________________________________________________________________________________________________________________________________________________________________________________________________________________________________________________________________
3. Supports coachee in identifying potential results or learning from identified action steps ________________________________________________________________________________________________________________________________________________________________________________________________________________________________________________________________________________________________________________________
4. Invites coachee to consider how to move forward, including resources, support and potential barriers ________________________________________________________________________________________________________________________________________________________________________________________________________________________________________________________________________________________________________________________
5. Partners with coachee to summarise learning and insight ________________________________________________________________________________________________________________________________________________________________________________________________________________________________________________________________________________________________________________________
6. Celebrates coachee’s progress and successes ________________________________________________________________________________________________________________________________________________________________________________________________________________________________________________________________________________________________________________________
7. Data Sources

*Reflection questions during coach training*

1. Reflecting on your current style of teaching, which of the 'coaching difficulties' might you be encountering more of? You will be exploring that during a practice coaching session.
2. Consider the steps of coaching mentioned in the article by Boyatzis. Please share your thoughts.
3. Do you see similarities and dissimilarities between the coaching process explained by Boyatzis and Gawande? Please elaborate
4. How does or does not the 'learning agenda' expressed by Boyatzis resonate with Ben Zander's 'A' student?
5. There is a scene that takes place in the woods, just off the fairway, where Junah is tempted to move the ball to make his swing easier. What might the implications of this scene be for coaching?
6. There is a scene that takes place in the woods, just off the fairway, where Junah is tempted to move the ball to make his swing easier. Can you think of a few questions a coach might ask a coachee?
7. How does the authentic swing described by Bagger Vance relate to coaching?
8. What lessons, either what you will emulate or what you will not emulate, can you draw from Bagger Vance in your journey to becoming an academic coach?
9. What makes Socratic questioning applicable in developing self-directed learning behavior?
10. What makes self-assessment an important skill in the development of a coach?
11. How would you rate yourself on your ability for self-assessment on a scale of 1 to 10, 1 being poor and 10 being excellent?
12. 'Different' learners are asking for the conversation about the 'difference'. Have you ever had conversations about race or other differences with the learners? What was that experience?
13. How would you rate your comfort level for coaching learners who identify themselves as members of an underrepresented minority group?
14. Coaching in medical education: What makes coaching, traditionally used in sports, music, and for executives, appropriate or inappropriate in the medical field?
15. Based on your reading of Ben Lovell's article, where do you envision yourself using coaching?
16. What challenges do you foresee in using coaching?

*Posttraining survey questions*

Q Thinking about **before** and **after** the program, please select the response that is most appropriate for you.

|  | Significantly increased | Somewhat increased | Neither increased nor decreased | Somewhat decreased | Significantly decreased |
| --- | --- | --- | --- | --- | --- |
| My understanding of coaching |  |  |  |  |  |
| My understanding of the difference between coaching, mentoring and advising |  |  |  |  |  |
| My attitude towards coaching |  |  |  |  |  |
| My belief in benefits of coaching |  |  |  |  |  |

Q How likely are you to recommend the Academic Coaching Program to a colleague?

- 0
- 1
- 2
- 3
- 4
- 5
- 6
- 7
- 8
- 9
- 10

Q Please select the most appropriate response for the following questions.

|  |  |  |  |  |  |
| --- | --- | --- | --- | --- | --- |
| Overall, I found the program valuable | - Strongly Agree | - Agree | - Neither agree nor disagree | - Disagree | - Strongly disagree |
| I had enough advance notice for the pre-work | - Strongly Agree | - Agree | - Neither agree nor disagree | - Disagree | - Strongly disagree |
| The pre-readings were thought-provoking and relevant | - Strongly Agree | - Agree | - Neither agree nor disagree | - Disagree | - Strongly disagree |
| The material covered during the program will be relevant and useful for my professional career | - Strongly Agree | - Agree | - Neither agree nor disagree | - Disagree | - Strongly disagree |
| The length of the program was just right | - Strongly Agree | - Agree | - Neither agree nor disagree | - Disagree | - Strongly disagree |
| The facilitators were well prepared | - Strongly Agree | - Agree | - Neither agree nor disagree | - Disagree | - Strongly disagree |
| Session time was used effectively | - Strongly Agree | - Agree | - Neither agree nor disagree | - Disagree | - Strongly disagree |
| Explanations were clear | - Strongly Agree | - Agree | - Neither agree nor disagree | - Disagree | - Strongly disagree |
| The course content was interesting | - Strongly Agree | - Agree | - Neither agree nor disagree | - Disagree | - Strongly disagree |

Q Thinking of the pre-work and readings between sessions, how much time did you spend to complete them?

- 1 hour
- 1.5 hours
- 2 hours
- 2.5 hours
- 3 hours
- 3.5 hours
- 4 hours
- >4 hours

Q Focusing on the coaching sessions, what is the **single most** valuable take-away for you from the program?

________________________________________________________________

Q Thinking of the coaching sessions what would you change in the next iteration?

________________________________________________________________

Q What ideas or thoughts would you like to share that is not already mentioned (may relate to content, material, presentation, presenters, planning for COM...)?

________________________________________________________________

________________________________________________________________

________________________________________________________________

________________________________________________________________

________________________________________________________________

Q Will you continue your development as a coach?

- Yes
- Maybe
- No

Skip To: Q12 If Will you continue your development as a coach? = No

Q How do you plan to continue with your development as a coach?

________________________________________________________________

Q12 Would you be interested in coaching sessions with observers once in six months or at a predetermined frequency?

- Yes
- Not sure
- No

*Focus Group Interview Protocol*

1. What is your view towards coaching after participating in this program?
   1. Follow-up: How have your views towards coaching after participating in this program been influenced?
2. How has the training program prepared you to coach medical students and residents?
3. What was most influential about participating in the training program?
4. How influential was reflection as a part of the training program?
   1. Follow-up: What method of reflection (e.g., face-to-face sessions, reading, discussions on Ment.io, small group sessions) used in this program was most helpful?
5. What aspect of the program would you enhance?
6. Were there other benefits to you from the program? If so, what are those?
7. Do you have any additional comments on the contents, teaching, and other materials related to the delivery of this program? If so, please share them.
